# 2642. Multipathogen Antigen Test Kit (MAK-5) – Distribution and Frequency of Viral Respiratory Diseases in Germany among Volunteers in the VACCELERATE Registry

**DOI:** 10.1093/ofid/ofad500.2254

**Published:** 2023-11-27

**Authors:** Julia A Nacov, Jon Salmanton-Garcia, Carolin Joisten, Christina Többen, Julian Fleig, Lisa Rochel, Louise Cremer, Zoi Dorothea Pana, Heinz-Josef Schmitt, Jannik Stemler, Oliver A Cornely

**Affiliations:** University Hospital Cologne, Cologne, Nordrhein-Westfalen, Germany; University Hospital Cologne, Cologne, Germany, Cologne, Nordrhein-Westfalen, Germany; University Hospital Cologne, Cologne, Nordrhein-Westfalen, Germany; University Hospital Cologne, Cologne, Nordrhein-Westfalen, Germany; University Hospital Cologne, Cologne, Nordrhein-Westfalen, Germany; University Hospital Cologne, Cologne, Nordrhein-Westfalen, Germany; University Hospital Cologne, Cologne, Nordrhein-Westfalen, Germany; European University of Cyprus, Nicosia, Nicosia, Cyprus; University Hospital Cologne, Cologne, Nordrhein-Westfalen, Germany; University Hospital Cologne, Cologne, Nordrhein-Westfalen, Germany; University of Cologne, Cologne, Germany, Cologne, Nordrhein-Westfalen, Germany

## Abstract

**Background:**

Currently, SARS-CoV-2 is the predominant viral respiratory pathogen. However, during winter season other viruses may cause acute respiratory infection (ARI). Most cases of non-severe ARI are not systematically tested, thus, distribution and epidemiology of these viruses is largely unknown.

VACCELERATE is the EU funded consortium dedicated to vaccine research, running a volunteer registry and promoting clinical studies.

**Methods:**

Adult registered subjects were invited to participate. Postal address was collected to allow sending of the MAK-5 test kit (BioTeke Corporation (Wuxi) Co., Ltd., Wuxi, Jiangsu, China). The availability of low-cost, sensitive and specific rapid tests detecting 5 pathogens (ADV, influenza viruses A and B, RSV, SARS-CoV-2) from one respiratory sample allowed us to conduct a feasibility study on their relative contribution to the burden of disease (BoD) outside any medical setting. Participants were instructed to use the test if respiratory symptoms were present for at least 24h. Test results and information on related symptoms and vaccinations were self-reported by e-mail. Underlying diseases were known from initial registration.

**Results:**

Between 07-Dec-2022 and 01-May-2023, we processed test results from 1036 (52.1%) of 1990 participants. Including co-infections (N=18; 4.7%), we detected 384 infections: 178 (46.4%) SARS-CoV-2, 59 (32.8%) 104 (27.1%) RSV and 74 (19.3%) influenza A virus. ADV infection was detected in 10 (2.6%) volunteers and influenza B virus in 6 (1.6%). A total of 98.8% of tests yielded valid results, while 12 (1.2%) tests were invalid (negative control). While RSV was the most frequently detected virus in the first week of assessment (calendar week (CW) 49), there was a shift to Influenza A virus in CW 50, followed by a SARS-CoV-2 peaks in CW 51/22, 05/23, and 08/23.

**Figure:**
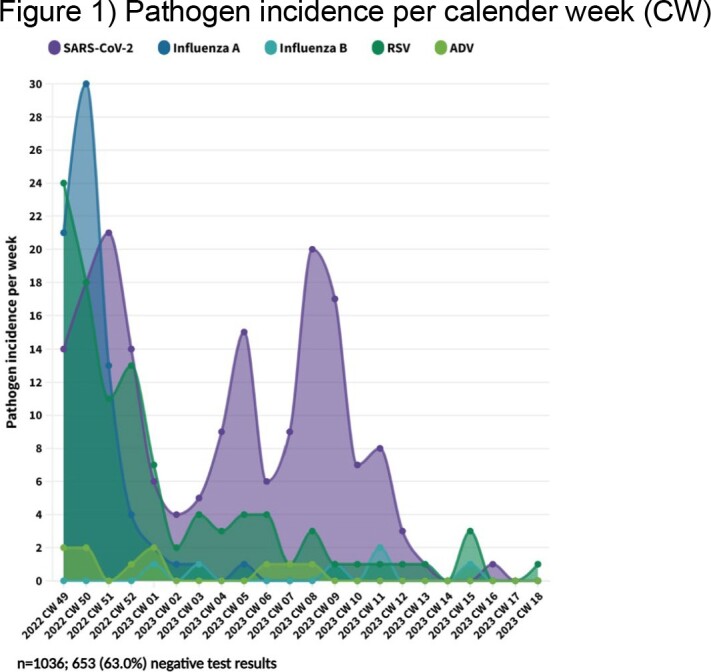

CW49/2022-CW18/2023

**Figure:**
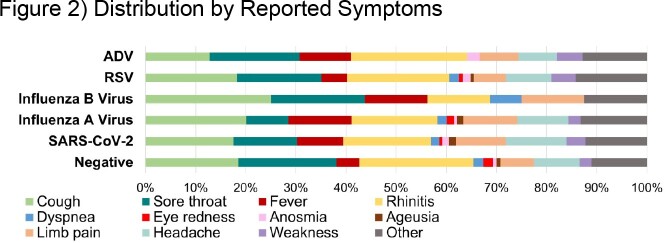

**Figure:**
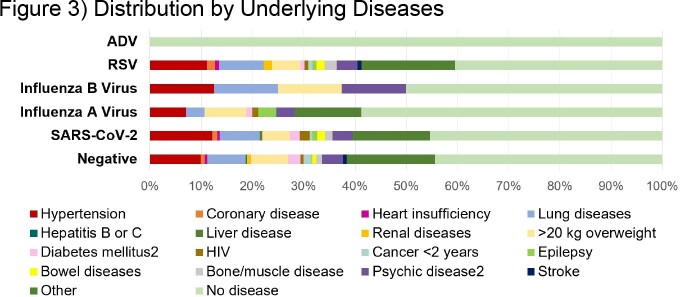

**Conclusion:**

This type of study allows assessment of the BoD by ARI pathogens before medical attention is sought and provides insights into the course of the annual (winter) infection waves down to a local level. Frequency of detected viruses varied over time. While SARS-CoV-2, RSV and influenza A virus were detected frequently, ADV and influenza B virus infections were rare.

**Disclosures:**

**Heinz-Josef Schmitt, n/a**, VACCELERATE: Advisor/Consultant **Jannik Stemler, -**, Ministry of Education and Research (BMBF): Grant/Research Support **Oliver A. Cornely, MD PhD**, DZIF: Advisor/Consultant|DZIF: Board Member|DZIF: Grant/Research Support|DZIF: Honoraria|DZIF: Stocks/Bonds

